# Personalized Disease Prevention (PDP): study protocol for a cluster-randomized clinical trial

**DOI:** 10.1186/s13063-022-06750-7

**Published:** 2022-10-22

**Authors:** Glen B. Taksler, Phuc Le, Bo Hu, Jay Alberts, Allen J. Flynn, Michael B. Rothberg

**Affiliations:** 1 Cleveland Clinic Community Care, Cleveland Clinic, 9500 Euclid Ave., G10, Cleveland, OH USA; 2 Department of Quantitative Health Sciences, Cleveland Clinic, Cleveland, OH USA; 3 Population Health Research Institute, Case Western Reserve University at The MetroHealth System, Cleveland, OH USA; 4 Department of Biomedical Engineering, Cleveland Clinic, Cleveland, OH USA; 5 Neurological Institute, Cleveland Clinic, Cleveland, OH USA; 6 School of Information and Department of Learning Health Sciences, University of Michigan, Ann Arbor, MI USA

**Keywords:** Preventive care, Medicine, Preventive, Preventive Health Services, Prevention, Primary, Disease prevention, Primary, Patient-specific modeling, Precision medicine

## Abstract

**Background:**

The US Preventive Services Task Force recommends 25 primary preventive services for middle-aged adults, but it can be difficult to do them all.

**Methods:**

The Personalized Disease Prevention (PDP) cluster-randomized clinical trial will evaluate whether patients and their providers benefit from an evidence-based decision tool to prioritize preventive services based on their potential to improve quality-adjusted life expectancy. The decision tool will be individualized for patient risk factors and available in the electronic health record. This Phase III trial seeks to enroll 60 primary care providers (clusters) and 600 patients aged 40–75 years. Half of providers will be assigned to an intervention to utilize the decision tool with approximately 10 patients each, and half will be assigned to usual care. Mixed-methods follow-up will include collection of preventive care utilization from electronic health records, patient and physician surveys, and qualitative interviews. We hypothesize that quality-adjusted life expectancy will increase by more in patients who receive the intervention, as compared with controls.

**Discussion:**

PDP will test a novel, holistic approach to help patients and providers prioritize the delivery of preventive services, based on patient risk factors in the electronic health record.

**Trial registration:**

ClinicalTrials.gov NCT05463887. Registered on July 19, 2022.

**Supplementary Information:**

The online version contains supplementary material available at 10.1186/s13063-022-06750-7.

In 2019, nearly two-thirds of US deaths were attributable to preventable risk factors [1, 2]. Despite 25 recommendations from the United States Preventive Services Task Force (USPSTF) for primary prevention in middle-aged adults [3], just 8% of adults ≥35 years received all high-priority services in 2015 [4]. Prevention is especially important during the COVID-19 pandemic, with evidence suggesting greater prevalence of alcohol misuse, weight gain, and lower dietary quality and physical activity than pre-pandemic [5–10].

Several obstacles limit primary care providers’ ability to deliver prevention effectively. First, preventive care is time-consuming. Discussing all guideline-recommended services with patients would take an estimated 7–9 h/day [11, 12], leaving little time for acute care needs. Second, evidence-based guidelines are written for broad populations rather than specific patients. Two-thirds of middle-aged adults have ≥1 comorbid condition [13], but the benefits of specific preventive services vary considerably across patients [14, 15]. Because there are no tools to calculate the benefits of a particular service for a particular patient, providers may have trouble communicating the relative benefit of different services [16]. Third, patients vary in their willingness to accept side effects, lifestyle changes, and medication costs, which may limit prevention effectiveness [3, 13, 17–19].

In prior work, we developed a mathematical model to individualize the benefits and harms of specific preventive services for patient risk factors [14, 15]. We collaborated with patients and providers to design a decision tool [20, 21] and pilot tested it with primary care patients [20]. Patients and providers found the tool helpful; results suggested potential improvement in patient knowledge, use of shared decision-making, readiness to change, and preventive care utilization [20].

The Personalized Disease Prevention (PDP) cluster-randomized clinical trial, designated Phase III by the National Institute on Aging, will assess whether an improved version of our individualized decision tool helps patients to improve their quality-adjusted life expectancy, as compared with usual care. PDP also will evaluate important prevention-related secondary outcomes, such as improved use of shared decision-making and readiness to change [14–16, 20, 21].

## Methods

The Personalized Disease Prevention (PDP) trial individualizes recommendations for preventive care and closely related chronic disease management services, based on 55 evidence-based risk factors. Each patient receives a different result based on his/her risk factors. Shown in a 1-page bar graph, patients may easily see which services are most likely to help them live a longer, healthier life. Providers may access the tool on-demand from the electronic health record (EHR) and are encouraged to discuss the individualized recommendations with patients using shared decision-making.

The foundation of the decision tool, called “individualized preventive care recommendations,” is a mathematical model developed by the study team. Previously published in proof-of-concept form [14, 15], the PDP model prioritizes 22 preventive and chronic disease management services based on their potential to improve a patient’s quality-adjusted life expectancy. Briefly, the model uses relative risk to adjust mean quality-of-life and survival probabilities (in 1-year increments) for the general population based on a patient’s age, sex, race, vitals, medical history, lifestyle, and family history. It then simulates the change in quality-of-life and survival probabilities achievable by following each preventive (or chronic disease management) service until the USPSTF-recommended stop age. For example, an individual who quits smoking would have lower risk of cardiovascular events, respiratory diseases, and various cancers, each of which would raise expected length and quality of life. Model parameters are derived from existing literature, often those referenced in evidence reviews or decision analyses accompanying USPSTF recommendations [14, 15]. It then rank-orders preventive services by the potential gain in quality-adjusted life years, to determine which services are most likely to help a patient live a longer, healthier life.

Leading up to this RCT, we worked with patients and providers to design the decision tool and then conducted a pilot study (*N*=104) [20, 21]. Patients randomized to receive individualized preventive care recommendations found the tool helpful (median rating by survey, 9/10) and wanted to use it again (10/10) [20]. Compared with controls, they demonstrated greater comprehension of the preventive (or chronic disease management) services most and least likely to improve their life expectancy and had greater mean improvement in shared decision-making, near-term readiness to change, and improvements in control of overweight/obesity, hypertension, hyperlipidemia, and diabetes [20].

### Study design

**Fig. 1:**
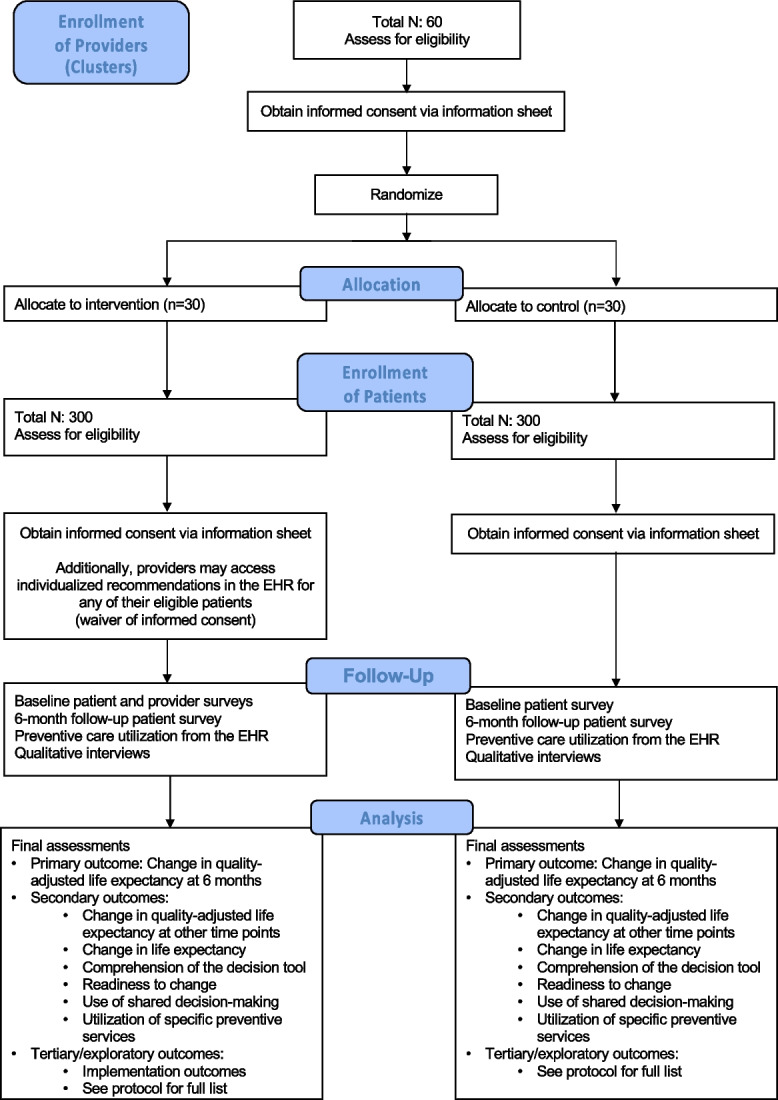
Overview of the Personalized Disease Prevention (PDP) cluster-randomized clinical trial. Brief overview (CONSORT-like diagram)

PDP is designed to allow evaluation of individualized preventive care recommendations in clinical practice, rather than a highly controlled setting. Table 1 shows a study timeline and Fig. 1 shows a CONSORT-like diagram. The study will be conducted at primary care sites within the Cleveland Clinic Health System (CCHS), which has a large academic medical center, 13 regional hospitals, 21 family health centers, and >75 outpatient locations. Despite its reputation for international referrals, 80% of primary care patients are from northeast Ohio. All Cleveland Clinic sites have shared a common EHR (Epic^TM^, Madison, WI) since 2006.

**Table 1:**
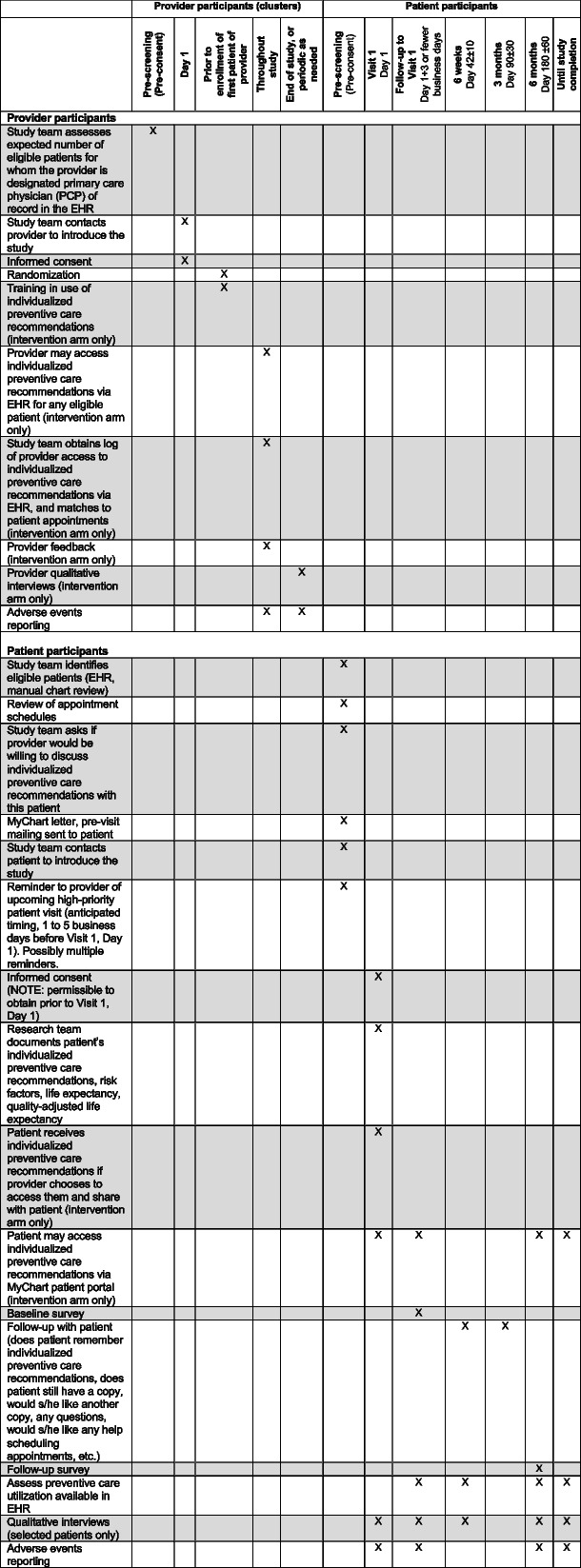
Study timeline. EHR: electronic health record

### Intervention

PDP will randomize providers (clusters) to receive access to individualized recommendations via the EHR for eligible patients (“intervention”) or usual care (“control”, chosen as a benchmark for current preventive care delivery). Trial design is a parallel, partially blinded, 1:1 allocation ratio. Intervention arm providers will be asked to access individualized recommendations and discuss them with patients. Consent will be via information sheet.

Additionally, utilizing a waiver of informed consent, providers will receive access to the EHR-based decision tool for all of their eligible patients, regardless of whether the study team follows them. We do so because providers may find it easier to remember that individualized recommendations are available for nearly all patients 40–75 years. Also, by encouraging providers to include this step in their clinical workflow, we will maximize chances for widespread adoption.

To accommodate clinical workflow, the model may be executed through a web app accessible in the EHR. Upon execution, the model’s inputs—a patient’s evidence-based risk factors—are automatically extracted from the EHR and individualized recommendations are output, typically within 3 s. The tool will be accessible in 1–2 clicks and will be updated each time the tool is opened. Providers may print the tool and copy/paste it to an after-visit summary (a post-encounter synopsis given to patients in normal workflow), which also is accessible to patients electronically from the health system’s patient portal.

### Preventive services included in the RCT

Table 2 describes the preventive services considered by the RCT and associated targets for eligible patients.

**Table 2:** Preventive and chronic disease management services included in the cluster-randomized clinical trial

Preventive service		Target
**Cancer screenings**		
1	Breast cancer^a^	Every 2 years
2	Cervical cancer	Co-testing (hrHPV testing plus cytology) every 5 years
3	Colorectal cancer	Decennial colonoscopy^b^
4	Lung cancer	Every 1 year
**Cardiovascular disease reduction**		
5	Abdominal aortic aneurysm screening	Once
6	Blood pressure control^c^	130/80 mmHg
7	Lipids control^c^	30% (low-intensity statins) or 50% (moderate-high intensity statins) reduction in LDL^d^
**Diabetes control**		
8	Diabetes control^c^	If baseline 7.0–7.9%, 8.0–8.9%, 9.0–10.9%, ≥11.0%: HbA1c = 1 point reduction, 7%, 2 point reduction, 9%, respectively
**Healthy lifestyle**^e^		
9	Alcohol misuse^c^	≤1 drink/day (female) or ≤2 drinks/day (male)
10	Bariatric surgery^c,f^	Mean of roux-en-Y gastric bypass and sleeve gastrectomy
11	Healthy diet	Lowest quintile of risk based on NHANES cycles 2013–2014 through 2017–2018
12	Light exercise	30 min per day
13	Moderate-vigorous exercise	150 min moderate or 75 min vigorous exercise per week, plus muscle strengthening exercise 2 days per week
14	Tobacco cessation^c^	Quit smoking
**Vaccines**		
15	Influenza vaccine^g^	Annual
16	Pneumonia vaccine^g^	PPSV23 (1–2 doses based on ACIP guidelines)
17	Tetanus vaccine^g^	Decennial
18	Zoster vaccine^g^	Two doses of Shingrix
**Other**		
19	Hepatitis C virus (HCV) testing^g^	Once
20	HIV testing^g^	Once (low-risk individuals) or annual (high-risk individuals)
21	Osteoporosis screening/falls prevention^g^	Once
22	Testing for sexually transmitted infections^g^	Annual in high-risk individuals

For each preventive service, the model defines eligibility based on the most recent USPSTF recommendation

*ACIP* Advisory Committee on Immunization Practices, *BMI* body mass index, *hrHPV* high-risk human papilloma virus, *RCT* randomized clinical trial, *USPSTF* United States Preventive Services Task Force

^a^The RCT excludes BRCA1/BRCA2 genetic testing and breast cancer chemoprevention, which are more relevant in younger women [22, 23] and often require specialist genetic counseling

^b^Annual fecal immunochemical testing is assumed to provide 90% of decennial colonoscopy benefit, based on a decision analysis accompanying the 2016 USPSTF recommendation [24]

^c^The RCT defines a target of risk factor control, rather than a USPSTF recommendation for screening or counseling. Diabetic foot exam is not included because it is expected to be routinely conducted at the baseline primary care visit for eligible patients, without need for shared decision-making. Diabetic eye exam is not included because many eligible Cleveland Clinic Health System patients obtain these exams from providers outside of the health system (e.g., opthamologist in private practice)

^d^Statin dosage will be assumed based on American College of Cardiology recommendations

^e^Depression screening not included because, typically, it would be faster to screen than to have a discussion about whether the screen a patient. Depression control not included because it is symptomatic; the focus of this RCT is primary prevention and asymptomatic chronic condition (or risk factor) control

^f^The USPSTF recommends weight loss counseling, which this RCT considers achievable through ≥1 of the following: bariatric surgery (assumed eligibility criteria: BMI≥40 kg/m^2^ or ≥35 kg/m^2^ in individuals with diabetes), healthy diet, and/or exercise. As with all services considered by the RCT, the individualized recommendations do not make a recommendation for or against receipt of bariatric surgery. The study assumes that a patient interested in bariatric surgery would have a discussion with his/her primary care provider and then a specialist. Additionally, the study team notes evolving evidence on medication (semaglutide) for weight loss, which may eventually be added to the RCT at the team’s discretion. The study team also may add a service Lose 10 lbs., intended to roughly proxy 5% weight loss, based on expected weight loss across available interventions (e.g., light exercise, partial adherence to healthy diet)

^g^Because the net benefit is likely to be small at the individual level (roughly, the public health benefit divided by the size of the at-risk population), the net benefit is assumed rather than mathematically modeled by the study team. For an average- or low-risk individual, typically assumed as ≤1 month of additional quality-adjusted life expectancy. Model documentation will provide further details, including definitions of high-risk factors and their individualized benefits (often, assumed as 1–2 months of additional quality-adjusted life expectancy)

### Example of individualized preventive care recommendations

**Fig. 2:**
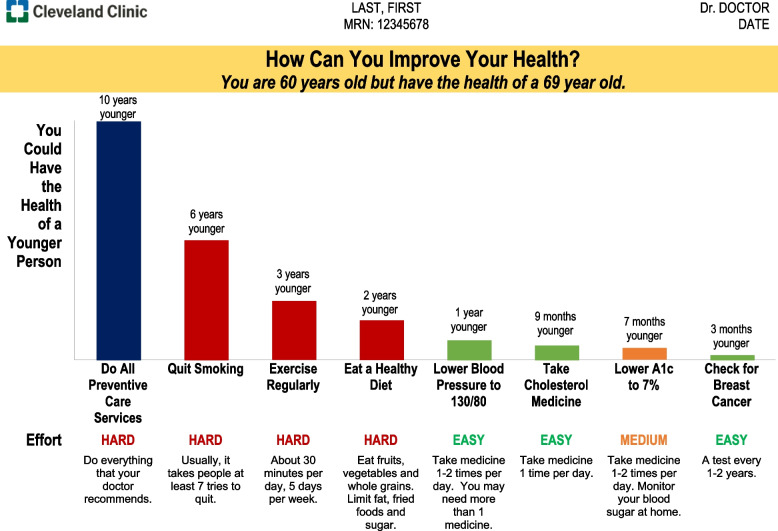
Example of decision tool shown to patients and providers. Color-coded bars indicate effort required to complete each preventive service or chronic disease management goal. Recommendations will be individualized for each patient’s age, sex, race, vitals, medical history, lifestyle, and family history, as reported in the electronic health record

To better understand the study, Fig. 2 provides an example of the decision tool that will be seen by a patient and provider. At top is an individualized statement; e.g., “You are 60 years old but have the health of a 69 year old.” Below, a bar graph shows the improvement if a patient utilizes all recommended preventive (and chronic disease management) services and that associated with each service, as previously described [20].

### Endpoints

Table 3 shows primary, secondary, and select tertiary study endpoints. The primary objective is to measure whether use of the tool improves patient quality-adjusted life expectancy (QALE), at a 6-month timeframe. We chose this outcome as an aggregate measure of utilization, alleviating the need to separately consider each service and to allow for the differential impact of each service on patient health (e.g., tobacco cessation vs. tetanus shot). For context, a 3-month increase in QALE is roughly equivalent to any of the following: lowering systolic blood pressure (BP) 5 mmHg, losing 5 lbs., low-dose statins, or both colorectal and breast cancer screenings. Actual magnitudes depend on each patient’s evidence-based risk factors, which vary substantially across patients.

**Table 3:** Study endpoints

Objectives	Endpoints	Hypothesis^**a**^
**Primary**		
To measure whether use of individualized preventive care recommendations is likely to help patients live a longer, healthier life	**Change in quality-adjusted life expectancy (QALE)** at 6 months, in patients whose providers are in the intervention arm, as compared with the control arm.^b^	Higher
**Secondary**		
To measure whether use of individualized preventive care recommendations is likely to help patients live a longer, healthier life	**Change in QALE** at each of the following time points: 12 months, all follow-up time points.	Higher
To measure whether use of individualized preventive care recommendations is likely to help patients live a longer life	**Change in life expectancy** (not quality-adjusted) at each of the following time points: 6 months, 12 months, all follow-up time points.	Higher
To assess comprehension of the decision tool	**Comprehension** of preventive services most likely to impact a patient’s quality-adjusted life expectancy, assessed by correct identification of each of the following: a. Service most likely to improve his/her QALE b. Service least likely to improve his/her QALE c. Correct identification of a patient’s true age (the age most commonly associated with his/her quality-adjusted life expectancy), in relation to his/her biological age	Higher
To assess readiness to change	Share of preventive services ready to change over the next 1 month, assessed by percent of patients with a mean score ≥6 on a 7-point scale for the (a) top-ranked and (b) bottom-ranked individualized preventive care recommendations.^b^	Higher
To assess use of use of shared decision-making	Use of shared decision-making (SDM), assessed by score on SDM-Q-9 survey [25, 26]	Higher
To assess utilization of specific services^c^	Change in weight, systolic BP, HbA1c, 10-year ASCVD risk score, LDL, total cholesterol, dietary quality (Starting the Conversation assessment) [27, 28], physical activity (modified International Physical Activity Questionnaire-Short Form) [29, 30], alcohol misuse (AUDIT-C) [31, 32], tobacco cessation; receipt of screening for cancers of the breast, cervix, colorectum, lung.	Improved (higher or lower depending on service)
**Select tertiary/exploratory**		
To assess reach	% of eligible patients for whom provider accesses individualized recommendations	None
To assess adoption	% of providers approached by the study team who agree to enroll; patient self-rating of: how helpful s/he found the recommendations, how interested s/he is in seeing individualized recommendations again in the future	None
To assess implementation	Adaptations made to intervention; known issues with fidelity	None
To assess maintenance	Provider reach at quarterly intervals post-enrollment; helpfulness of individualized recommendations 6 months after enrollment, self-reported by patient survey.	None

This table shows primary, secondary, and select tertiary/exploratory study endpoints. See the study protocol for all tertiary/exploratory endpoints

^a^ In patients of intervention arm providers, as compared with patients of control arm providers

^b^ “Top-” (“bottom-”) ranked individualized preventive care recommendations are defined as follows: top (bottom) 3 for patients with ≥6 recommendations, 2 for patients with 4–5 recommendations, 1 for patients with 3 recommendations, not applicable for patients with ≤2 recommendations. Only collected for preventive services that a patient states his/her provider discussed during the baseline encounter

^c^ Assessed for the subgroup of patients recommended each service. Only considered when follow-up data are available for ≥30 high patients of intervention arm providers and ≥30 patients of control arm providers

Because an increase in QALE may be difficult to achieve, we will assess a number of important secondary outcomes. Disease prevention is a complex process, requiring behavior change at the patient, provider, health system, and state/national levels. We will learn whether the tool promoted desirable changes for preventive care and chronic disease management: comprehension of the tool, use of shared decision-making, and readiness to change. If comprehension and/or shared decision-making are high, then even if patients do not ultimately change their preventive care (or chronic disease management), they better understand their health care needs, resulting in a more informed decision. If readiness to change is high, then the tool may have helped patients want to utilize preventive (or chronic disease management) services, but additional interventions are needed to improve adherence and QALE.

### Inclusion criteria

#### Providers

Eligible providers will be any attending physician, nurse practitioner, or physician assistant practicing in internal medicine or family medicine.

#### Patients

Eligible patients will have the following inclusion criteria:

1. Aged 40–75 years.
2. A modifiable lifestyle factor with a large impact on QALE, assessed by ≥1 of the following: current smoker, body mass index (BMI) ≥30.0 kg/m^2^, BP ≥140/90 mmHg, 10-year atherosclerotic cardiovascular disease (ASCVD) risk ≥10%, glycated hemoglobin (HbA1c) ≥9% or alcohol consumption/week of >7 drinks (4.2 oz) for females or >14 drinks (8.4 oz) for males. Our rationale is that ≥1 individualized recommendation should have a high magnitude of impact on quality-adjusted life expectancy. Consistent with primary prevention, alcohol misuse will focus on asymptomatic excess; e.g., 2–3 drinks most nights without dependency.
3. Eligible for a high number of preventive services, assessed by ≥3 of the following: current smoker, BMI ≥27.0 kg/m^2^﻿, systolic BP >130 mmHg, 10-year ASCVD risk ≥7.5%, HbA1c ≥7.5%, alcohol consumption/week of >7 drinks (4.2 oz) for females or >14 drinks (8.4 oz) for males, overdue/due soon for colorectal cancer screening, overdue/due soon for lung cancer screening, overdue for ≥1 year for breast cancer screening, overdue for ≥1 year for osteoporosis screening. Our rationale is that patients with a high number of individualized recommendations may have greater need for prioritization, as compared with other patients.
4. Ongoing primary care in the health system, defined as ≥2 in-person or virtual visits with a primary care provider (PCP) in the prior 730 days. Our rationale is that follow-up EHR data are more likely to exist for patients with ongoing primary care, as compared with other patients.
5. Annual wellness visit or closely related encounter (e.g., hypertension follow-up) with the patient’s PCP of record. Virtual visits are eligible, although in-person visits may be preferable.

### Exclusion criteria

Exclusion criteria will include conditions that severely limit life expectancy, necessitate secondary prevention, or symptomatically alter the need for primary prevention; or limited-no ability to communicate in English. The full study protocol (current version 1.2 [July 7, 2022]) provides details.

### Criteria for inclusion in the EHR

Individualized recommendations will be included in the EHR (for on-demand provider access) for any patient aged 40–75 years with a PCP assigned to the intervention arm, who does not meet above exclusion criteria.

### Intervention

Patients decide which preventive (and chronic disease management) services to pursue in complex environments, influenced by the desire to improve their health and other factors (e.g., personal obligations at work or at home) that affect feasibility. By better understanding which preventive (and chronic disease management) services are most likely to promote a longer, healthier life, we hypothesize that patients can improve their health outcomes.

**Fig. 3:**
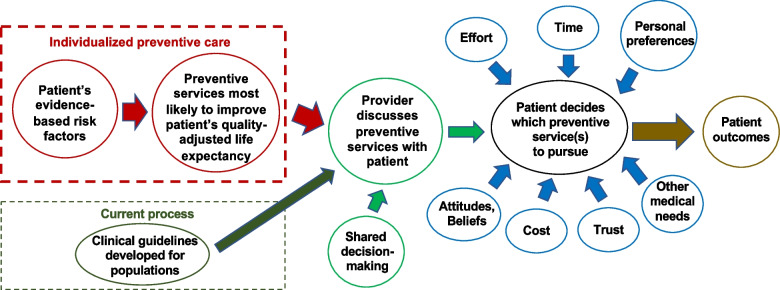
Conceptual framework

Figure 3 describes the study’s conceptual framework, guided by the heterogeneity of treatment effect (HTE) model [33–35]. HTE addresses variation in a study’s results across individual patients, who, based on their individual risk factors, derive different benefit from an intervention. Few patients receive the “average” treatment effect. Patients at high-risk benefit more, while those at low-risk may not benefit at all. Here, we quantify the net benefits of various preventive and chronic disease management interventions for individual patients based on their evidence-based risk factors. We convert these to a single metric, the change in QALE from utilizing each service, and rank-order the results. This contrasts with a current process of clinical guidelines developed for *populations* of patients. Next, a provider will discuss the individualized recommendations with a patient. Rather than a sequential approach (e.g., hypertension control, then glycemic control, then breast cancer screening), we will ask providers to engage in a holistic conversation about all of a patient’s primary prevention (and chronic disease management) needs utilizing shared decision-making. A patient has *options* to improve his/her health, none of which is required, but all of which would be beneficial. The patient must decide which service(s) to pursue, based on our model and external factors: effort (e.g., lifestyle changes are difficult); available time in the context of work, family, hobbies; personal preferences; attitudes/beliefs; cost; trust in the provider and health system; and other medical needs not addressed by our intervention. We hypothesize that patient outcomes will improve if providers help patients understand which preventive (and chronic disease management) services are most likely to improve their QALE, and discuss them using shared decision-making, as compared with current processes. Below, we describe the intervention in detail.

#### Providers

Through departmental staff meetings and direct invitations, the study team will contact eligible providers. Those who consent (via information sheet) will be randomized. Intervention arm providers will be invited to a 1-h training on the study, use of individualized preventive care recommendations and shared decision-making [36–39]. Those who complete the training will receive access to individualized preventive care recommendations in the EHR.

#### Patients

Through automated data feeds from the EHR linked with upcoming appointment schedules, and manual chart review as needed, the study team will identify specific patients. We will ask if the patient’s provider is willing to discuss individualized recommendations with this patient. The team will seek to enroll the patient and remind providers shortly before the scheduled encounter.

### Mixed-methods feedback

Patients will be asked to complete two 15–20-min surveys, within 3 business days after the baseline encounter and 6 months later. Surveys will inform overall impressions, select study endpoints and suggestions for future work (Table 4). At 6 weeks and 3 months post-encounter, a team member will call patients to ask if they need another copy of the individualized recommendations or have questions. Providers will be asked to complete a short survey, within 3 business days after each baseline patient encounter. Also, we will conduct qualitative interviews of patients and providers, approximately quarterly, and request regular (informal) provider feedback (Table 4). Qualitative feedback will inform whether the tool is something that patients and providers *want* to use. ﻿Given time constraints in primary care and countless alert messages in the EHR, the tool is only likely to help patients if providers proactively open it in the EHR and discuss with patients. Finally, throughout the study, we will assess preventive (and chronic disease management) service utilization documented in the EHR, which will inform study endpoints. Surveys and qualitative interviews will utilize gift card incentives to promote retention.

**Table 4:** Patient and provider feedback

Patients			Providers	
Baseline survey	6 m survey	Qualitative interviews	Regular, informal feedback	Qualitative interviews
**Overall impressions**				
Use of individualized preventive care recommendations during encounter Helpfulness of individualized preventive care recommendations (intervention only) (Likert scale)	Usefulness of individualized preventive care recommendations	Did your doctor talk with you about preventive care during your appointment? Did your doctor give you any written information? What is your opinion of it? How did this visit compare with other visits you have had with your doctor?	What did you like about the tool? Dislike? How often do you use the tool? Why (why not)?	What value did the decision tool add to patient encounters? What is an example of when the tool enhanced communication about preventive care? When it did not help?
**Use of shared decision-making**				
Use of shared decision-making (SDM-Q-9) [25, 26]	-	How did your doctor involve you in that conversation? What was most helpful? What could be improved?	Would you please tell me a bit about how you discuss the tool with patients?	Did the tool encourage shared decision-making? Please explain.
**Study endpoints**				
Comprehension of preventive service most likely and least likely to improve QALE^a^ Readiness to change (transtheoretical model) [40] Lifestyle^b^	Self-reported preventive (and chronic disease management) service utilization Lifestyle^b^	-	-	-
**Future directions**				
Interest in using individualized preventive care recommendations again in the future (Likert scale)			Suggestions for improvement	Would you like to keep using the tool? Are there obstacles?
**Other**				
Demographics Self-rated health			How well did the intervention fit with your clinical workflow? How can we improve workflow?	Patients for whom the tool was particularly helpful (not helpful) What other information would be helpful?

^a^ Left- and right-hand bars in Fig. 2

^b^ Tobacco use, alcohol (AUDIT-C) [31, 32], healthy diet (Starting the Conversation) [27, 28], physical activity (IPAQ-SF) [29, 30]

### Adverse events (harms)

As a minimal risk study, safety events are unlikely. However, because individualized recommendations may upset patients, we will measure anxiety/depression, defined as a new mental health diagnosis, score on the Generalized Anxiety Disorder[GAD]-7 questionnaire≥8 or Patient Health Questionnaire[PHQ]-8≥10 (asked in patient surveys) [41, 42]. Additionally, we also collect data on harms of preventive services that patients may choose to undergo (or not) due to the intervention, but it is very unlikely these would be study-related (full study protocol).

### Randomization

Provider randomization will be stratified by site (1:1 allocation ratio) in permuted block sizes of 2, 4, and 6. The study biostatistician will conceal the block sizes and generate the sequence with computer-generated random numbers. Study staff who enroll providers will not have access to the sequence; instead, they will communicate enrollment to designated study coordinators.

### Blinding

Because the nature of PDP requires frequent interaction with providers (e.g., intervention feedback), cluster randomization will be unblinded. However, the Principal and Co-Investigators, except the study biostatistician and safety assessor, will be blinded to stratification of outcomes and safety events by arm. The DSMB may request unblinding of specific participants.

### Collection of study endpoints

The primary outcome, change in patient QALE between the baseline encounter and 6 months later, and most secondary outcomes will be estimated through our mathematical model. Inputs will be obtained from an EHR data feed for each patient, from the baseline encounter through study completion. Select lifestyle endpoints with limited-no availability in the EHR (healthy diet, physical activity), wide variance in documentation quality (alcohol misuse) or that may have been changed since the last update in the EHR (tobacco) may be self-reported using validated scales in patient surveys [27–32]. The full study protocol specifies an objective algorithm in case EHR and self-reported data conflict.

### Statistical analysis

The biostatistician will conduct analyses based on an equivalence design, with a modified intention-to-treat (ITT) patient population. Each patient who completed the baseline primary care encounter (excluding no shows, cancellations, etc.) and his/her provider will be treated in the group according to initial randomization. Linear mixed-effect models with random intercepts at the patient and provider levels will be used. Such models assume data missing at random. Sensitivity analyses may consider full ITT and per protocol populations, as well as multiple imputation for missing data. Full ITT is not expected to yield significant results because approx. 1/3 of primary care patients do not show up or cancel scheduled encounters [20]. Subgroup analyses will be by race. There are no planned interim analyses.

### Power

A sample size of 60 providers (30/arm), each with 10 patients, will have 86.2% power to detect a clinically meaningful 3.0-month difference in the change of QALE between the 2 arms. We assume a small intra-cluster correlation coefficient (ICC)=0.01 because life expectancies of different patients are unlikely to be correlated, even from the same provider. In sensitivity analysis, estimated power is 80.4% for a sample size of 54 providers and an ICC=0.02. Therefore, the study will still have adequate power if 10% of patients are lost to follow-up, consistent with our pilot study [20].

### Data management

Study data will be entered and stored in RedCap (Nashville, TN).

### Oversight

Cleveland Clinic’s Institutional Review Board (IRB) approved this study and considers it minimal risk. The National Institute on Aging (NIA) approved the protocol, members of an independent Data and Safety Monitoring Board (DSMB), and a DSMB charter. The IRB, NIA, and DSMB must approve protocol amendments and may specify terms for communication of amendments to trial participants.

## Discussion

PDP will be the first Phase III RCT to test the impact of an individualized decision tool to prioritize the delivery of nearly all major preventive services and closely rated chronic disease management services. Middle-aged patients are asked to adhere to up to 25 preventive service recommendations [43], a tall order for even the most motivated patients. Instead, they must prioritize based on factors including effort, available time, and acute medical needs, and even providers must prioritize discussion of evidence-based recommendations based on available time and perceived importance [11, 12, 16, 21]. PDP hypothesizes that patient outcomes will improve through a holistic understanding of which services are most likely to promote healthy aging, discussed with providers using shared decision-making.

This trial will expand prior literature in 3 dimensions. First, PDP will employ a mathematical model to translate evidence-based benefits and harms of preventive (and chronic disease management) services into a single metric, quality-adjusted life expectancy. Prior work focused on length, not quality, of life [20, 44, 45]. In prior work, both patients [21] and physicians [16] reported quality-of-life as a highly relevant metric by which to prioritize delivery of preventive services. Through this process, the resulting decision tool will offer a magnitude of benefit and rank-order, so that patients may better understand the relative importance of various recommendations.

Second, PDP will rigorously test a novel approach to decision aids, which seek to improve risk communication and shared decision-making [20, 46–48]. Whereas most tools consider single decisions; e.g., whether to take statins [49, 50], PDP will simultaneously address all evidence-based preventive services in an easy-to-follow 1-page bar graph [20]. Providers will be asked to spend their usual amount of time discussing preventive care, but reorganize the discussion holistically.

Third, model inputs will be obtained automatically from the EHR, alleviating the need for time-intensive manual data entry. This step should greatly improve the chances of reaching less motivated patients, who may be unwilling to complete a questionnaire with model inputs. This decision necessitates a provider-, rather than patient-facing tool in prior work [51], because EHR patient portals (which are accessed externally—outside a firewall) have less advanced capabilities than a clinician-facing EHR. We will do so in a generally scalable approach, which should facilitate eventual dissemination to other health systems, particularly those utilizing Epic^TM^’s EHR [52].

We note several limitations. First, even if the intervention increases QALE, there is potential for decrease in utilization of select preventive (or chronic disease management) services. For example, we may find that the intervention promotes tobacco cessation but reduces cancer screening utilization or vaccine uptake (because of lower expected benefits). Such a result would increase QALE—and therefore healthier aging, consistent with PDP’s objectives—but also result in some potential harm. Second, mathematical models are by nature imperfect. With DSMB approval, our model targets +/− 10–20% error compared with published population/policy-level models [53–60]. This is consistent with studies finding that patients better comprehend the gist (a central message, presented visually) than an exact magnitude [46, 61]. Third, the model’s recommendations will only be as good as EHR documentation quality. Previously, we developed and validated an EHR-based primary care registry of >800,000 primary care patients, giving confidence that overall documentation is excellent [62]. However, certain inputs (e.g., alcohol misuse, family history) are less-well documented [62]. Providers will be trained accordingly. Should the intervention prompt them to improve documentation, this limitation would become a strength.

In conclusion, the PDP cluster-randomized trial offers a rigorous design for evaluating the effect of individualized preventive service (and chronic disease management) recommendations on patient outcomes in routine primary care.

## Supplementary Information

**Additional file 1.**

## Data Availability

De-identified participant data, subject to compliance with organizational policies; local institutional review board rules; local, state and federal laws and regulations, including the HIPAA Privacy Rule; and the NIH data sharing policy.
