# Supplementary material for: Personalized Disease Prevention (PDP): study protocol for a cluster-randomized clinical trial
Source: Trials. 2022 Oct 22;23:892. doi: 10.1186/s13063-022-06750-7 (PMC9587586; doi:10.1186/s13063-022-06750-7)
Supplement: Supplementary file 1 — Additional file 1. [file 13063_2022_6750_MOESM1_ESM.pdf]

Patient Study Information Sheet

Project Title: Personalized Disease Prevention (PDP): a randomized clinical trial
Principal Investigator: Glen Taksler, PhD (216-445-7499)

Your health care provider is participating in a research study about different ways to discuss health with patients. At your visit, your provider may show you some materials and discuss them with you. If so, the materials were created *just for you* based on your health needs and risk factors. No other patient will receive the same materials.

We would like to invite you to participate in the research study as well. If you agree, we will ask you to complete 2 surveys and possibly participate in an interview.

If you choose to participate, the study team will access your medical records in the future to evaluate the impact of the research study on health outcomes.

If you are interested in participating, this information sheet gives you information about the study. The study staff can review this information with you. The study staff will explain the purpose of the study, any risks to you and what is expected of you. You are free to ask questions about the study at any time. This study has been approved by Cleveland Clinic's Institutional Review Board. Before you learn more about the study, it is important for you to know that your participation in this study is entirely voluntary. You may decide not to take part in, or to withdraw from, the study at any time.

Why are you being asked to participate in this study?
You are being asked to participate because your health care provider is also participating in a research study.

What is the purpose of this study?
The purpose of this study is to help patients learn the benefits of various preventive care services (things you can do to prevent future health care problems). Your provider may show you written or electronic materials created just for you. We call this a decision tool.

What are the requirements and time commitment to participate?
You may be asked to participate in a survey after your visit. You may also be contacted about 6 months later to complete another survey. We may also contact you to ask about your visit or experience with the research study.

Also, if you are interested, we may ask you to participate in a 30-60 minute telephone interview about the research study. You don't have to do the interview unless you want to. Even if you are interested, we may not ask because we only need to interview a small number of patients.

If you choose to participate, the study team will access your medical records in the future to evaluate the impact of the research study on health outcomes. This will help us find out whether

different ways to discuss health care eventually change patients' health.

What are the benefits to taking part in the study?
There is no direct benefit to you. Participating in this study may help researchers gain further knowledge about the best ways to discuss preventive care services with patients.

Are there any risks to you in participating in the study?
There are no physical risks associated with the study. Some of the questions asked as part of the survey may make you feel uncomfortable. You may refuse to answer any of the questions. There is also the possibility that the survey questions and/or interview could cause psychological stress or fatigue to you. You may stop the survey and/or interview at any time.

There is a potential risk of loss of confidentiality of your data. Data will be stored in password-protected and encrypted storage facilities accessible only by the research team. Participant names will be replaced by a study number so that individuals who do not need to know your name will not be able to connect your name with the research data. Personal information that could identify you will be removed or changed before files are shared with other researchers or results are made public. However, federal or state laws or regulations may require us to show information to people responsible for monitoring the safety of this study, such as government officials (or sponsors) or their designees (for example, members of a data and safety monitoring board required by the National Institutes of Health).

Also, if you choose to receive electronic (digital) gift cards, we will share your email address with a web site that is not part of Cleveland Clinic. If you do not want us to share your email address, please choose a physical gift card.

If you choose to participate in an interview, the interview may be transcribed (put into writing) by a third-party who agrees to maintain confidentiality.

Do I have to participate in the survey?
Your participation is strictly voluntary. Your decision to participate in the survey will not impact your current or future medical care at Cleveland Clinic. You may choose not to take part or may stop the survey at any time. Stopping the survey and/or interview will not result in any penalty.

What other options are there?
Your participation is strictly voluntary. The alternative is to not participate.

What are the costs?
There are no costs for you to participate.

If you choose to participate in the surveys, you will receive a $40 gift card for completion of each survey (total $80).

If you choose to participate in the interview, you will receive another $40 gift card (total $120).

If you prefer, a paper check can be issued rather than a gift card.

The IRS requires CCF to report payments to an individual of $600 or greater (in a calendar year) on a Form 1099-MISC. Your name, address and social security number will be collected to track the payments made to you and, if you receive $600 or greater, will be used to process a Form 1099-MISC.

Who do I contact if I have questions about the study?
If you have questions about the study after you have agreed, you may ask the study coordinators Jackie Fox, RN (216-444-4590), Holly Renner, RN (440-684-8900) or Toyomi Goto, MA (216-444-8424) for assistance at any time or you can contact Glen Taksler, PhD (principal investigator) at 216-445-7499. If you have any questions about your rights as a research subject, you may contact the Institutional Review Board at (216) 444-2924.

Patient Study Information Sheet

Project Title: Personalized Disease Prevention (PDP): a randomized clinical trial
Principal Investigator: Glen Taksler, PhD (216-445-7499)

Your health care provider is participating in a research study about different ways to discuss health with patients.

We would like to invite you to participate in the research study as well. If you agree, we will ask you to complete 2 surveys.

If you choose to participate, the study team will access your medical records in the future to evaluate the impact of the research study on health outcomes.

If you are interested in participating, this information sheet gives you information about the study. The study staff can review this information with you. The study staff will explain the purpose of the study, any risks to you and what is expected of you. You are free to ask questions about the study at any time. This study has been approved by Cleveland Clinic's Institutional Review Board. Before you learn more about the study, it is important for you to know that your participation in this study is entirely voluntary. You may decide not to take part in, or to withdraw from, the study at any time.

Why are you being asked to participate in this study?
You are being asked to participate because your health care provider is also participating in a research study.

What is the purpose of this study?
The purpose of this study is to help patients learn the benefits of various preventive care services (things you can do to prevent future health care problems).

What are the requirements and time commitment to participate?
You may be asked to participate in a survey after your visit. You may also be contacted about 6 months later to complete another survey. We may also contact you to ask about your visit or experience with the research study.

If you choose to participate, the study team will access your medical records in the future to evaluate the impact of the research study on health outcomes. This will help us find out whether different ways to discuss health care eventually change patients' health.

What are the benefits to taking part in the study?
There is no direct benefit to you. Participating in this study may help researchers gain further knowledge about the best ways to discuss preventive care services with patients.

Are there any risks to you in participating in the study?
There are no physical risks associated with the study. Some of the questions asked as part of the survey may make you feel uncomfortable. You may refuse to answer any of the questions. There is also the possibility that the survey questions could cause psychological stress or fatigue to you. You may stop the survey at any time.

There is a potential risk of loss of confidentiality of your data. Data will be stored in password-protected and encrypted storage facilities accessible only by the research team. Participant names will be replaced by a study number so that individuals who do not need to know your name will not be able to connect your name with the research data. Personal information that could identify you will be removed or changed before files are shared with other researchers or results are made public. However, federal or state laws or regulations may require us to show information to people responsible for monitoring the safety of this study, such as government officials (or sponsors) or their designees (for example, members of a data and safety monitoring board required by the National Institutes of Health).

Also, if you choose to receive electronic (digital) gift cards, we will share your email address with a web site that is not part of Cleveland Clinic. If you do not want us to share your email address, please choose a physical gift card.

Do I have to participate in the survey?
Your participation is strictly voluntary. Your decision to participate in the survey will not impact your current or future medical care at Cleveland Clinic. You may choose not to take part or may stop the survey at any time. Stopping the survey will not result in any penalty.

What other options are there?
Your participation is strictly voluntary. The alternative is to not participate in the survey.

What are the costs?
There are no costs for you to participate.

If you choose to participate in the surveys, you will receive a $40 gift card for completion of each survey (total $80). If you prefer, a paper check can be issued rather than a gift card.

The IRS requires CCF to report payments to an individual of $600 or greater (in a calendar year) on a Form 1099-MISC. Your name, address and social security number will be collected to track the payments made to you and, if you receive $600 or greater, will be used to process a Form 1099-MISC.

Who do I contact if I have questions about the study?
If you have questions about the study after you have agreed, you may ask the study coordinators Jackie Fox, RN (216-444-4590), Holly Renner, RN (440-684-8900) or Toyomi Goto, MA (216-444-8424) for assistance at any time or you can contact Glen Taksler, PhD (principal investigator) at 216-445-7499. If you have any questions about your rights as a research subject, you may contact the Institutional Review Board at (216) 444-2924.

The Cleveland Clinic Foundation
Consent to Participate in an Interview for a Research Study

Project title: Personalized Disease Prevention (PDP): a randomized clinical trial
Sponsor: National Institutes of Health
Principal Investigator: Glen Taksler, PhD (216-445-7499)
Study Coordinators: Jackie Fox, RN (216-444-4590), Holly Renner, RN (440-684-8900) and Toyomi Goto, MA (216-444-8424)
After hours phone contact #: Call 216-444-2200 or 1-800-223-2273 and ask for the Internal Medicine physician on-call

KEY INFORMATION

You are a participant in a research study by Cleveland Clinic.
The project title is "Personalized Disease Prevention (PDP): a randomized clinical trial". By participating in this study, you help Cleveland Clinic researchers understand different ways to discuss health with patients.

What am I being invited to do today?
You are being invited to complete an interview. It will take about 30 to 60 minutes.

Do I have to participate in the interview?
Your participation is strictly voluntary. You do not have to participate the interview, and will remain part of the research study even if you do not do the interview.

Do I have to continue participating in the research?
Your participation is strictly voluntary. You can withdraw at any time.

<u>The following is a short summary of the interview part of the research study to help you decide whether or not to participate. More detailed information is included later on in this document.</u>

What should I know about the interview part of the research study?
- Someone will explain this part of the research study to you.
- You can choose whether or not to take part.
- You can agree to take part and then later change your mind.
- Your decision whether or not to participate will not be held against you.
- You can ask all the questions you want before you decide.

What is the purpose, procedures and duration of the interview part of the study?

The purpose of this study is to help patients learn the benefits of various preventive care services (things you can do to prevent future health care problems). At a recent appointment, your provider showed you written or electronic materials created just for you. We call this a decision tool.

To help us understand if the decision tool was helpful, we are seeking patient feedback.

You will be asked to participate in a 30 to 60 minute audio-recorded interview.

Your participation in the interview will last about 30 to 60 minutes.

More detailed information can be found under the section labeled: "Information on the Research."

Why might you choose not to participate in the interview part of the research study?

You might choose not to participate in this research study because some of the questions asked as part of the interview may make you feel uncomfortable or cause psychological stress or fatigue to you. There is a potential risk of loss of confidentiality of your data (information about you).

More detailed information about the risks of this study can be found in the section labeled "Risks."

Why might you choose to volunteer for the interview part of the research study?

Participating in the interview part of the research study may help researchers learn more about the best ways to discuss preventive care services with patients.

More detailed information about the benefits of this study can be found in the section labeled "Benefits."

What are my other choices if I do not take part in the interview part of the research study?
The alternative is to not participate.

More detailed information about the alternatives to this study can be found in the section labeled "Alternatives."

DETAILED INFORMATION

The following is more detailed information about this study in addition to the information listed above.

1. INFORMATION ON THE RESEARCH

Why is the research study being done?

The primary objective of this study is to help patients learn the benefits of various preventive care services (things you can do to prevent future health care problems). At your recent

appointment, your provider showed you written or electronic materials created just for you. We call this a decision tool.

To help us understand if the decision tool was helpful, we are seeking patient feedback. We plan to interview patients to get their feedback.

How many people will take part in the interview part of the research study?

About 40 patients will take part (participate in an interview) in this study at Cleveland Clinic.

What is involved if you decide to take part in the interview part of the research study?

A member of the study team will conduct an in-depth, audio-recorded private interview. You may do the interview over the phone or in-person, whichever you prefer.

2. ALTERNATIVES

What are the alternatives to participation in the interview part of the research study?

Your participation is strictly voluntary. The alternative is to not participate.

3. RISKS

What are the risks of participating in the research study?

There are no physical risks associated with the study. Some of the questions we will ask you as part of this study may make you feel uncomfortable. You may refuse to answer any of the questions. There is also the possibility that the interview questions could cause psychological stress or fatigue to you. You may stop the interview at any time.

You may refuse to answer any of the questions and you may take a break at any time during the study. You may stop your participation in this study at any time.

<u>Confidentiality Risks</u>

There is a potential risk of loss of confidentiality of your data. Every effort will be made to keep your information confidential through the use of the following safeguards: All data will be stored in password-protected and encrypted storage facilities accessible only by the research team. Participant names will be replaced by a study number so that individuals who do not need to know your name will not be able to connect your name with the research data.

Your interview will be audio recorded for purposes of transcription (writing) which may be needed for research analysis. Your audio recording will not be disclosed outside of Cleveland Clinic for purposes other than transcription. Only de-identified (anonymous) text quotes will appear in manuscripts or publications; your audio recording (voice) will not be presented publicly. The study team may include quotations from your interview in research publications and presentations, but the quotations will not include any information that could identify you.

Also, if you choose to receive electronic (digital) gift cards, we will share your email address with a web site that is not part of Cleveland Clinic. If you do not want us to share your email address, please choose a physical gift card.

4. BENEFITS
What are possible benefits of participating in the interview part of the research study ?

There is no direct benefit to you. Participating in this study may help researchers gain further knowledge about the best ways to discuss preventive care services with patients.

5. COSTS
Are there any costs to you if you participate in this study?

There is no cost to you to be in this research study.

6. PAYMENT
Are there any payments to you if you participate in this study?

If you choose to participate in the interview, you will receive a $40 gift card. If you prefer, a paper check can be issued rather than a gift card.

The IRS requires CCF to report payments to an individual of $600 or greater (in a calendar year) on a Form 1099-MISC. Your name, address and social security number will be collected to track the payments made to you and, if you receive $600 or greater, will be used to process a Form 1099-MISC.

7. RESEARCH RELATED INJURY
What will happen if you are injured as a result of taking part in the research?

The potential for research related injury is rare as this study involves interviews.
You are not waiving any legal rights by signing this form. Further information about research related injury is available by contacting the Institutional Review Board at (216) 444-2924.

8. PRIVACY AND CONFIDENTIALITY

What will happen to your information that is collected for this research?

Cleveland Clinic may share your study information, without anyone knowing that it is related to you specifically, with others or use it to research projects not listed in this form. Your data may be stored and shared for future research without additional informed consent if identifiable private information, such as your name and medical record number, are removed. If your

identifying information is removed from your data, we will no longer be able to identify and destroy them.

Study results may be shared in medical journals, at scientific meetings, and in other mediums without your identifying information. Your records will be confidential and your identity will not be shared in medical journals, at scientific meetings, and in other mediums without your express consent.

Authorization to Use/Disclose Protected Health Information

Cleveland Clinic has rules and procedures to protect information about you. Federal and State laws also protect your privacy.

The research team working on the study will collect information about you. This includes your health information, data collected for this research study and personal identifying information including your name, address, date of birth and other identifying information.

Generally, only people on the research team will know your identity and that you are in the research study. However, sometimes other people at Cleveland Clinic may see or give out your information. These include people who review research studies including the Institutional Review Board and Research Compliance, their staff, lawyers, or other Cleveland Clinic staff.

People outside Cleveland Clinic may see your information for this study. Examples include government groups (such as the Food and Drug Administration), safety monitors, other hospitals in the study and the sponsor of the research and their agents. Cleveland Clinic will do our best to ensure your information is kept confidential and that only the health information which is minimally required to conduct the study is used or disclosed to people outside Cleveland Clinic; however, people outside Cleveland Clinic who receive your information may not be covered by this promise.

Consistent with federal regulations, authorized representatives of the National Institutes of Health (NIH) and an external Data Safety Monitoring Board, whose members are not employed by Cleveland Clinic, may have the right to periodically review your study data. The NIH requires us to make study data available to researchers at other institutions for use in future research, using a process specifically designed for this purpose called a data repository, which is not owned by Cleveland Clinic. Before doing so, we will remove any information that identifies you (other than your study number) so that individuals using the data will not know who you are.

You do not have to give this permission to use and give out your information; however, you will not be able to participate in this research study without providing this permission by signing this consent form. The use and disclosure of your information has no expiration date.

You may cancel your permission to use and disclose your information at any time by notifying the Principal Investigator in writing:
Glen Taksler, PhD
Cleveland Clinic
9500 Euclid Avenue

Cleveland, Ohio 44195

If you do cancel your permission to use and disclose your information, your participation in this study will end and no further information about you will be collected. Your cancellation would not affect information already collected in the study.

Clinical Trials Language

A description of this clinical trial will be available on http://www.ClinicalTrials.gov, as required by U. S. law. This Website will not include information that can identify you. At most, the Website will include a summary of the results. You can search the Website at any time.

9. QUESTIONS

Who do you call if you have any questions or problems?

If you have any questions or concerns about the research, or develop a research-related problem, you should contact Glen Taksler PhD (principal investigator) at (216-445-7499) or study coordinators Jackie Fox, RN (216-444-4590), Holly Renner, RN (440-684-8900) and Toyomi Goto, MA (216-444-8424) for assistance at any time. During non-business hours, weekends and holidays, please contact call 216-444-2200 or 1-800-223-2273 and ask for the Internal Medicine physician on-call. If you have questions about your rights as a research subject, you should contact the Institutional Review Board at (216) 444-2924.

10. VOLUNTARY PARTICIPATION

What are your rights as a research participant?

Taking part in this study is voluntary. You will be told of any new, relevant information from the research that may affect your health, welfare, or willingness to continue in this study. You may choose not to take part or may leave the study at any time. Withdrawing from the study will not result in any penalty or loss of benefits to which you are entitled. If you decide to withdraw from the study you should discuss with your study doctor your decision to ensure a safe withdrawal.

If you leave the study early, Cleveland Clinic may use or give out your health information that it has already collected if the information is needed for this study or any follow-up activities.

11. SIGNATURES

Statement of Participant

I have read and have had verbally explained to me the above information and have had all my questions answered to my satisfaction. I understand that my participation is voluntary and that I may stop my participation in the study at any time. Signing this form does not waive any of my legal rights. I understand that a copy of this consent will be provided to me. By signing below, I agree to take part in this research study.

____
Printed name of Participant

_______________________________ ____________
Participant Signature Date

Statement of Person Conducting Informed Consent Discussion

I have discussed the information contained in this document with the participant and it is my opinion that the participant understands the risks, benefits, alternatives and procedures involved with this research study.

____
Printed name of person obtaining consent

_______________________________ ____________
Signature of person obtaining consent Date

USE THIS WHEN A WITNESS IS USED IN THE CONSENTING PROCESS (Common examples include: Inclusion of illiterate individuals, blind individuals or individuals who cannot physically sign but are able to provide informed consent and/or Remote Consent/Greater than minimal risk Studies.)

_____________________________ _______________________ ___________
Signature of Witness Printed Name of Witness Date

Employee Study Information Sheet

Project Title: Personalized Disease Prevention (PDP): a randomized clinical trial
Principal Investigator: Glen Taksler, PhD (216-445-7499)

Thank you for taking the time to review this information.

This information sheet gives you information about the study which the study staff will then review with you. The study staff will explain the purpose of the study, any risks to you and what is expected of you. You are free to ask questions about the study at any time. This study has been approved by Cleveland Clinic's Institutional Review Board. Before you learn more about the study, it is important for you to know that your participation in this study is entirely voluntary. You may decide not to take part in, or to withdraw from, the study at any time.

Why are you being asked to participate in this study?
You are being asked to participate in this study because you are a health care provider at Cleveland Clinic in adult primary care services.

What is the purpose of this study?
This study is a randomized clinical trial. The primary objective is to evaluate a decision tool, created by the study team and available in Epic™, called *individualized preventive care recommendations*. These recommendations provide a best guess of the impact of major preventive services on a patient's health, individualized for his/her age, sex, race, medical history, lifestyle and family history as documented in Epic™. The process uses evidence-based medicine to take a best guess of what US Preventive Services Task Force (USPSTF) and Advisory Committee on Immunization Practices (ACIP) recommendations would have been, had they been created just for your patient.

What are the requirements and time commitment to participate?
You will be asked to complete a 1-minute enrollment survey (e.g., years in practice, preferred method of communication, etc.). Then, you will be randomized to either an "intervention" or "control" arm. <u>If randomized to the intervention</u>, then you will be asked to participate in training about the preventive care recommendations and shared decision-making. Several options for training sessions will be provided and if needed, and we will block your schedule to prevent conflicting patient appointments.

We will enable a new screen in your Epic™ account, providing access to individualized preventive care recommendations for the vast majority of your patients aged 40-75 years.

We will ask you to regularly access the individualized preventive care recommendations in Epic™, especially for your patients with extensive health maintenance needs or risk factors (e.g., smoking; obesity; uncontrolled diabetes, hypertension or hyperlipidemia). We strongly encourage you to use shared decision-making to facilitate the discussion.

Additionally, based on criteria reviewed by the National Institutes of Health, the study team may designate some of your patient encounters (typically about 10) as "high priority." For each high priority patient encounter, the study team plans to contact you in advance and specifically ask you to discuss the individualized recommendations. You may agree or decline to do so. If you agree, then we may ask you to complete a short online survey after the encounter. We also may ask the patient to complete a survey shortly after the visit and about 6 months later, contact him/her from time-to-time, and monitor his/her subsequent attainment of preventive services (based on EHR data and a follow-up patient survey[s]).

Your patients may be able to access their individualized preventive care recommendations in MyChart, and we send them a hard copy (printout) by mail.

To facilitate feedback, we have designated (or will designate) several provider champions. We ask you to regularly tell us your comments and suggestions, both positive and negative. You also may be asked to participate in a qualitative interview.

To analyze the trial, we will document your access to individualized recommendations in Epic™. We also will compare attainment of preventive services in your high priority patients after introduction of the tool in Epic™, as compared with before introduction of the tool. These analyses are solely intended to evaluate the success or failure of the tool in helping to improve preventive care.

We anticipate that your time commitment will be as follows:

1. the first few times you try the tool, about 10 minutes to discuss the individualized recommendations with your patient
2. afterwards, roughly your usual amount of time discussing preventive care, but organized as a holistic discussion rather than sequential discussion of preventive services
3. regular feedback may take 5-10 minutes in the beginning and 2-3 minutes ongoing, per week
4. online surveys may take 3 minutes each
5. randomized trial qualitative interviews may last up to 45 minutes

<u>If you are randomized to the control arm</u>, then will you will be asked to continue with usual care. However, based on criteria reviewed by the National Institutes of Health, the study team may designate some of your patient encounters (typically about 10) as "high priority." For each high priority patient encounter, the study team may ask the patient to complete a survey shortly after the visit and about 6 months later, and monitor his/her subsequent attainment of preventive services (based on EHR data and a follow-up patient survey[s]). The study team will not inform you in advance which patient(s) are designated high priority. After an encounter, the study team may inform you, but only if you specifically request such information, and you must make this request after each encounter of interest.

What are the benefits to taking part in this study?

You may be able to help improve patient adherence to preventive care recommendations. An earlier pilot study, conducted at 3 Cleveland Clinic primary care sites, found that patients who discussed individualized preventive care recommendations with their providers were more likely

to lose weight and better control hypertension, hyperlipidemia and diabetes over the next year, as compared with patients who received usual care. The study was too small to detect use of other preventive services. Additionally, participating in this study may improve knowledge about social determinants of health that limit use of preventive care.

Are there any risks to you in participating in this study?
There are no physical risks associated with this study. If discussion of the written materials takes longer than anticipated, you may be late for other patient appointments. Some of the survey and/or qualitative interview questions may make you feel uncomfortable or cause psychological stress or fatigue. You may refuse to answer any of the questions.

There is a potential risk of loss of confidentiality of your data. Data will be stored in password-protected and encrypted storage facilities accessible only by the research team. Participant names will be replaced by a study number so that individuals who do not need to know your name will not be able to connect your name with the research data. Personal information that could identify you will be removed or changed before files are shared with other researchers or results are made public. However, federal or state laws or regulations may require us to show information to people responsible for monitoring the safety of this study, such as government officials (or sponsors) or their designees (for example, members of a data and safety monitoring board required by the National Institutes of Health).

Also, if you choose to receive electronic (digital) gift cards, we will share your email address with a web site that is not part of Cleveland Clinic. If you do not want us to share your email address, please choose a physical gift card.

If you choose to participate in a qualitative interview, the interview may be transcribed (put into writing) by a third-party who agrees to maintain confidentiality.

No one with direct supervisory responsibilities for your work will have access to your individual responses. Results will be reported in aggregate and will not be linked to specific providers.

Do I have to participate in the research?
Your participation is strictly voluntary. Your decision to participate will not impact your current or future employment a Cleveland Clinic or your performance reviews. The decision to participate or not to participate in our study will not affect your employment status in any way.

You may choose not to take part or may leave the study at any time. Leaving the study will not result in any penalty.

What other options are there?
Your participation is strictly voluntary. The alternative is to not participate.

What are the costs?
There is no cost for participation.

If you are randomized to the intervention and choose to participate in the provider training, you will receive a $75 gift card. If you choose to participate in the online survey after high-priority patient encounters, you will receive a $10 gift card for each completed survey. If you choose to participate in the qualitative interview, you will receive a $50 gift card (total $225 if you complete 10 online surveys).

Per Cleveland Clinic Tax Department guidelines, gift cards are considered taxable wages and will be reported on your pay stub. For this purpose only, your name and employee ID will be provided to the payroll department.

Who do I contact if I have questions about the research?
If you have questions about the study after you have agreed, you may ask the study coordinators for assistance at any time: Jackie Fox, RN (216-444-4590), Holly Renner, RN (440-684-8900), Toyomi Goto, MA (216-444-8424) for assistance at any time or you may contact Glen Taksler, PhD (principal investigator) at 216-445-7499. If you have any questions about your rights as a research subject, you may contact the Institutional Review Board at (216) 444-2924.

The first time you access individualized recommendations in Epic™, provide the study's electronic or written materials to a patient, discuss the electronic or written materials with a patient, provide after-visit feedback to the research team, and/or complete a study survey and/or qualitative interview, the activity will indicate your agreement to participate in the research study. Additionally, each time you conduct any of these activities will indicate your continued consent to participate in the study.

Version 3/8/22
